# Supplementary material for: Discrete two dimensional Fourier transform in polar coordinates part II: numerical computation and approximation of the continuous transform
Source: PeerJ Comput Sci. 2020 Mar 2;6:e257. doi: 10.7717/peerj-cs.257 (PMC7924731; doi:10.7717/peerj-cs.257)
Supplement: Supplemental Information 1 [file peerj-cs-06-257-s001.docx]

# Appendix: Sample Matlab code for the discrete 2D Fourier transform in polar coordinates

## A-1. Theta matrix for space limited function

Reviewer Hans Feichtinger is acknowledged for the suggested code modifications

% N1 sample size in radial direction
% N2 sample size in angular direction
function theta=thetamatrix_SpaceLimited(N2,N1)
b2 = 2*pi/N2;
progr2= -pi + b2/2 : b2 : pi - b2/2;
theta = progr2(:)*ones(1,N1-1);

## A-2. r matrix for space limited function

% N1 sample size in radial direction
% N2 sample size in angular direction
% R effective space limit
% zeromatrix is precalculated Bessel zeros
function r=rmatrix_SpaceLimited(N2,N1,R,zeromatrix)
M=(N2-1)/2;
for ii=1:N2;
 p=ii-1-M;
 for k=1:N1-1;
 zero2=zeromatrix(5001-abs(p),:);
 jpk=zero2(k);
 jpN1=zero2(N1);
 r(ii,k)=(jpk/jpN1)*R;
 end
end

## A-3. Psi matrix for space limited function

% N1 sample size in radial direction
% N2 sample size in angular direction
function psi=psimatrix_SpaceLimited(N2,N1)
b2 = 2*pi/N2;
progr2= -pi + b2/2 : b2 : pi - b2/2;
psi = progr2(:)*ones(1,N1-1);

## A-4. Rho matrix for space limited function

% N1 sample size in radial direction
% N2 sample size in angular direction
% R effective space limit
% zeromatrix precalculated Bessel zeros
function rho=rhomatrix_SpaceLimited(N2,N1,R,zeromatrix)
M=(N2-1)/2;
for ii=1:N2;
 q=ii-1-M;
 for l=1:N1-1;
 zero2=zeromatrix(5001-abs(q),:);
 jql=zero2(l);
 rho(ii,l)=jql/R;
 end
end

## A-5. Y matrix Assembly Function

% Y is the N-1 x N-1 transformation matrix to be assembled
% n is the order of the bessel function
% N is the size of the transformation matrix
%zeros are the bessel zeros passed to the function

function Y = YmatrixAssembly(n,N,zero)
%tic


for l=1:N-1

 for k=1:N-1

 jnk=zero(k);
 jnl=zero(l);
 jnN=zero(N);
 jnplus1=besselj(n+1, jnk);

 Y(l,k)=(2*besselj(n,(jnk*jnl/jnN)))/(jnN*jnplus1^2);


 end
end

%toc

end

## A-5. Forward transform of Gaussian function

N2=15; %number of sample points in angular direction
N1=383; %number of sample points in radial direction
M=(N2-1)/2; %highest order of bessel function
R=40;% space limit
Wp=30; % band limit
a=0.1;
load('zeromatrix.mat')
theta=thetamatrix_SpaceLimited(N2,N1); %Sample point in angular direction in space domain.
r=rmatrix_SpaceLimited(N2,N1,R,zeromatrix);%Sample point in radial direction in space domain.
psi=psimatrix_SpaceLimited(N2,N1);%Sample point in angular direction in frequency domain.
rho=rhomatrix_SpaceLimited(N2,N1,R,zeromatrix);%Sample point in radial direction in frequency domain.
[x,y]=pol2cart(theta,r); %sample points in Cartesian coordinates in space domain
[x1,y1]=pol2cart(psi,rho); %sample points in Cartesian coordinates in frequency domain


%Discretizing the function
gau = @(x) exp(-(x).^2);
f=gau(r);

% DFT
fnk=circshift(fft(circshift(f,M+1,1),N2,1),-(M+1),1);
% DHT
for n=-M:M
 ii=n+M+1;
 zero2=zeromatrix(5001-abs(n),:);
 jnN1=zero2(N1);
 if n<0
 Y=((-1)^abs(n))*YmatrixAssembly(abs(n),N1,zero2);
 else
 Y=YmatrixAssembly(abs(n),N1,zero2);
 end
 fnl(ii,:)=(Y*fnk(ii,:)')';
 Fnl(ii,:)=fnl(ii,:)*(2*pi*(i^(-n)))*(R^2/jnN1);
end
% IDFT
TwoDFT=circshift(ifft(circshift(Fnl,M+1,1),N2,1),-(M+1),1);
%creating a discrete 2D Fourier transform
gau2 = @(x) pi*exp((-x.^2)/4);
trueFunc=gau2(rho);


%calculating the dynamic error from transform and true function
error= 20*log10(abs(trueFunc- TwoDFT)/max(max(abs(TwoDFT))));

figure(1)
subplot(2,1,1)
surf(x1,y1,abs(trueFunc))
title('\fontsize{24}Sampled Continuous Forward Transform')
subplot(2,1,2)
surf(x1,y1,abs(TwoDFT))
title('\fontsize{24}Discrete Forward Transform')

figure(2)

surf(x1,y1,error)
xlabel('x');
ylabel('y');
zlabel('db')
str=sprintf('Error distribution with N2 = %d, N1 = %d,R= %d, a= %d ', N2,N1,R,a);
title(['\fontsize{24}Error distribution with N2=',num2str(N2),', N1=',num2str(N1),', R=',num2str(R), ', Wp=',num2str(Wp)]);

mean1=mean(mean(error)); % Average dynamic error
max1=max(max(error)); % Maximum dynamic error

## A-6. Inverse transform of Gaussian function

N2=15 ; %number of sample points in angular direction
N1=383; %number of sample points in radial direction
M=(N2-1)/2; %highest order of bessel function
R=40;% space limit
Wp=30; % band limit
a=0.1;
load('zeromatrix.mat')
theta=thetamatrix_SpaceLimited(N2,N1);%Sample point in angular direction in space domain.
r=rmatrix_SpaceLimited(N2,N1,R,zeromatrix);%Sample point in radial direction in space domain.
psi=psimatrix_SpaceLimited(N2,N1);%Sample point in angular direction in frequency domain.
rho=rhomatrix_SpaceLimited(N2,N1,R,zeromatrix);%Sample point in radial direction in frequency domain.
[x,y]=pol2cart(theta,r); %sample points in Cartesian coordinates in space domain
[x1,y1]=pol2cart(psi,rho); %sample points in Cartesian coordinates in frequency domain

%creating a discrete true function
gau2 = @(x) pi*exp((-x.^2)/4);
trueFunc=gau2(rho);

% DFT
FNL=circshift(fft(circshift(trueFunc,M+1,1),N2,1),-(M+1),1);
% DHT
for n=-M:M
 ii=n+M+1;
 zero2=zeromatrix(5001-abs(n),:);
 jnN1=zero2(N1);
 if n<0
 Y=((-1)^abs(n))*YmatrixAssembly(abs(n),N1,zero2);
 else
 Y=YmatrixAssembly(abs(n),N1,zero2);
 end
 Y0=Y';
 Fnk(ii,:)=FNL(ii,:)*Y0;
 fnk(ii,:)=Fnk(ii,:)*((jnN1)*(j^n))/(2*pi*R^2);

end
% IDFT
TwoDIFT=circshift(ifft(circshift(fnk,M+1,1),N2,1),-(M+1),1);

%%discretizing the function in space domain
gau = @(x) exp(-(x).^2);
f=gau(r)


%calculating the dynamic error from transform and origal function
error= 20*log10(abs(f- TwoDIFT)/max(max(abs(TwoDIFT))));

figure(1)
subplot(2,1,1)
surf(x,y,abs(f))
title('\fontsize{24}Continuous Inverse Transform')
subplot(2,1,2)
surf(x,y,abs(TwoDIFT))
title('\fontsize{24}Discrete inverse Transform')

figure(2)
surf(x,y,error)
xlabel('x');
ylabel('y');
zlabel('db')
str=sprintf('Error distribution with N2 = %d, N1 = %d,R= %d, a= %d ', N2,N1,R,a);
title(['\fontsize{24}Error distribution with N2=',num2str(N2),', N1=',num2str(N1),', R=',num2str(R), ', Wp=',num2str(Wp)]);


mean=mean(mean(error)); % Average dynamic error
max=max(max(error));% Maximum dynamic error
